# Linking the Dynamic Changes in the In Vitro Antioxidant Activity of Carob Kibbles upon Roasting to the Chemical and Structural Changes Revealed by FTIR Spectroscopy

**DOI:** 10.3390/antiox10122025

**Published:** 2021-12-20

**Authors:** Anna Marina Grigoriou, Eftychia Pinakoulaki

**Affiliations:** Department of Chemistry, University of Cyprus, Nicosia 1678, Cyprus; grigoriou.anna-marina@ucy.ac.cy

**Keywords:** carobs, polyphenolic extracts, radical scavenging activity, Maillard reaction products, melanoidins, ATR-FTIR spectroscopy

## Abstract

Recent studies have highlighted the potential of utilizing carob kibbles as a bioactive-rich food ingredient associated with substantial health benefits. Roasting is a key process in enhancing the sensory characteristics of carob kibbles, also affecting the bioactive polyphenols and leading to the formation of Maillard reaction products (MRPs), including the polymeric melanoidins that are associated with a high antioxidant potential but remain unexplored in carob. In this work, we employed for the first time attenuated total reflectance-Fourier Transform Infrared (ATR-FTIR) spectroscopy to probe the dynamic chemical and structural changes upon the roasting of carob kibbles, along with the investigation of the in vitro antioxidant activity through the 2,2-Diphenyl-1-picrylhydrazyl (DPPH) radical scavenging activity and the determination of the total polyphenolic, proanthocyanidin, gallic acid and cinnamic acid contents. Roasting significantly enhanced the in vitro antioxidant activity of the polyphenolic carob extracts, with different rates at distinct roasting temperatures. The ATR-FTIR analysis enabled the identification of the changes in the structural features of polyphenolic compounds that were related to the improved antioxidant activity upon roasting. Furthermore, the detection of characteristic signatures for the polymeric melanoidins in the infrared (IR) fingerprint region provided the first evidence for the formation and structural properties of these complex, diverse compounds in roasted carob kibbles.

## 1. Introduction

Carob is the fruit of an evergreen tree (*Ceratonia siliqua* L.) cultivated mainly in the Mediterranean area [1]. Although carob is a valuable source of dietary fibers, sugars, minerals and a range of bioactive compounds, including polyphenols, its use in the food industry has been largely limited to its seeds, until recently [2,3]. The seeds represent about 10% of the carob pod weight and are utilized for the production of the valuable locust bean gum (LBG), which is a natural food additive functioning as a thickener, stabilizer and flavorant [4]. The separation of the seeds from the carob pod leaves the pulp, comprising 90% of the pod, as a by-product known as carob kibbles. During the last years, there has been a growing number of studies and reviews that highlight the potential of utilizing carob kibbles as a bioactive-rich food ingredient for the production of new food products, confectionaries and beverages [2,5,6,7]. The chemical composition of carob is determined by several factors, including variety, climatic and agronomic conditions, the ripening stage and can also be significantly influenced by processing procedures, such as roasting [8,9]. The roasting of carob kibbles is implemented in order to improve sensory characteristics, namely taste, color and aroma, especially in the production of carob powder, a substitute and extender for cocoa [2]. In the conventional roasting of carob kibbles, a temperature range of 120–180 °C is used for 10–60 min, followed by milling and, depending on the roasting parameters, lightly, medium or dark roasted carob powder with cocoa-like sensory attributes is produced. Studies on the impact of roasting have been increasing during the last years and are mainly focused on the sensory, physical and functional attributes, the phenolic content and antioxidant activity or formation of contaminants in carob powder [10,11,12,13,14,15]. However, a comprehensive picture of the changes involving bioactive antioxidant compounds upon the roasting of carob kibbles, is still lacking.

One class of compounds that has been the focus of several studies regarding carob, is polyphenols [16,17,18,19,20]. Polyphenols are phytochemicals that contribute to the improvement of human health and prevention of numerous diseases, including cardiovascular diseases, oxidative stress and cancer, and exhibit neuroprotective properties due to their antioxidant and anti-inflammatory activities [21]. Carob polyphenolic extracts have been demonstrated to display anti-proliferative and apoptotic activity against cancer cells, in addition to anti-diabetic, anti-diarrheal and anti-hyperlipidemic effects [22,23,24,25,26,27]. The major polyphenols present in carob include free, bound and conjugated forms of phenolic acids, flavonols, flavan-3-oles, hydrolysable tannins and proanthocyanidins with significant biological activities [2,17,23,24,26]. The polyphenolic content of carob powder can be influenced through competing pathways upon roasting, as the thermal degradation of phenolic compounds can lead to decreased antioxidant activity, while higher extractability of phenolic compounds from the complex food matrix upon heating has been suggested to increase availability and, consequently, to induce the opposite effect on antioxidant activity [11,12,17]. Although the antioxidant properties of carob powder are largely attributed to its polyphenolic content, chemical reactions that impact antioxidant activity occur upon roasting, with the Maillard reaction (the reaction between reducing sugars and amine groups of amino acids) being a major contributor, as carob pulp has a high sugar content (40–55%) and appreciable amount of protein (2–7%) [2]. The Maillard reaction products (MRPs) include a wide variety of compounds, such as intermediate reactive α-dicarbonyls, heterocycles, reductones and the high molecular weight polymeric nitrogen-containing melanoidins [28,29]. Even though melanoidins are widely distributed in thermally processed foods, such as cocoa, bakery products, coffee, cooked meat and potatoes, their chemical structure is complex, diverse and, in the present time, actively being investigated. Their biological effects are generally considered as positive and include antimicrobial and antioxidant activity associated with their ability to chelate metals, scavenge radicals and decompose hydrogen peroxide [28,29]. Interestingly, the presence of bound phenolics in the high molecular weight melanoidins has been described in roasted coffee and cocoa [30,31,32]. Melanoidins produced in carob upon roasting have not yet been studied and their structural features remain elusive.

Considering the novel aspects of the utilization of carob kibbles, it is imperative to understand the chemical changes taking place during roasting, in particular regarding potential bioactive compounds, namely polyphenols and MRPs. In the present study, we utilized attenuated total reflectance Fourier Transform Infrared (ATR-FTIR) spectroscopy, a method that is sensitive to molecular structure, to probe the dynamic chemical and structural changes in carob powder extracts upon the roasting of kibbles at different temperatures and roasting times. Moreover, the 2,2-Diphenyl-1-picrylhydrazyl (DPPH) radical scavenging activity was employed to evaluate the antioxidant activity of the corresponding extracts from raw and roasted carob kibbles, together with the determination of the total polyphenolic content (TPC), proanthocyanidin, gallic acid and cinnamic acid contents, to provide quantitative information on polyphenolic compounds.

## 2. Materials and Methods

### 2.1. Chemicals and Standards

Folin–Ciocalteu’s phenol reagent, gallic acid (>97.5%); sodium carbonate (>99.0%); 2,2 -diphenyl-1-picryl hydrazyl (DPPH); methanol (gradient grade for LC); 6-hydroxy-2,5,7,8-tetramethylchroman-2-carboxylic acid (97%; trolox); ethanol (gradient grade for LC), n-butanol, hydrochloric acid (37%); ammonium iron(III) sulfate dodecahydrate (>99%); (+)-catechin hydrate (≥98%); (−)-epicatechin (≥90%); caffeic acid (≥98.0%); trans-ferulic acid (99%); trans-cinnamic acid (≥99%); quercetin (≥95%); acetic acid (>99.5%) and acetonitrile (gradient grade for LC) were provided by Sigma-Aldrich (Milan, Italy). Pyrogallol (>99%), trans-p-coumaric acid (>98%), myricetin (>97.0%) and kaempferol hydrate (>97.0%) were supplied by TCI (Tokyo Chemical Industry, Tokyo, Japan). Cyanidin chloride was provided by the European Pharmacopoeia Reference Standard (Strasbourg, France).

Ultrapure water was prepared in a Milli-Q filter system (Millipore, Milan, Italy).

### 2.2. Plant Material and Roasting

Carob pods were obtained from local producers in Paphos, Cyprus and stored at room temperature. The pods were first washed with water and air dried. After removing the seeds, the pods were kibbled at a thickness of 0.5 to 1.0 cm and roasted in a pre-heated oven at different temperature/time conditions: at 125 °C (15, 30, 45 and 60 min); at 150 °C (15, 30, 45 and 60 min) and at 175 °C (15, 30 and 45 min). For the temperature of 175 °C, roasting for 60 min resulted in partial carbonization and the formation of undesirable organoleptic characteristics, thus, this sample was rejected. Roasting experiments were performed in duplicate. After roasting, samples were ground in a Thermomix TM5 (Vorwerk, Berkshire, UK) until powdered (carob powder).

### 2.3. Extraction Method

Polyphenolic extracts were obtained according to the method described by Kumazawa et al., with modifications [16]. Carob powder was extracted with cold water and allowed to stand for 12 h at 4 °C, followed by filtration to remove sugars. The residue was then extracted with 50:50 ethanol:water for 10 min with reflux. The sample was allowed to stand for 30 min at room temperature and filtered. The filtrate was then centrifuged for 10 min at 10,000 rpm at 4 °C using an Eppendorf 5804R centrifuge with a F-34-6-38 rotor (Hamburg, Germany), concentrated at the R-215 rotary evaporator (Büchi Flawil, Switzerland) to remove ethanol and freeze dried at −55 °C, 0.1 mbar using a LyoDry Compact Benchtop Freeze Dryer (Mechateck Systems, Bristol, UK). The lyophilized material was stored at 4 °C for up to 4–5 days until analysis. Extraction experiments were performed in duplicate.

### 2.4. Determination of Total Polyphenolic Content

The total polyphenolic content of the extracts was determined using the Folin–Ciocalteu photometric method, as described by Uysal et al., with some modifications [33]. Gallic acid was used as the standard for the calibration curve. Standard solutions at concentrations of 0.05–0.75 mg/mL were prepared. A total of 4.5 mL distilled water, 100 μL of the extract (2 mg/mL concentration) or the standard solution and 100 μL Folin–Ciocalteu reagent were added to a vial and the vial was shaken vigorously. After 3 min, 300 μL of Na_2_CO_3_ 2% solution was added and the mixture was allowed to react for 2 h. The absorbance was measured at 760 nm using a Shimadzu UV-1700 spectrometer (Tokyo, Japan). The total polyphenolic content was expressed as mg gallic acid equivalents per gram of extract dry weight (mg GAE/g dw).

### 2.5. Determination of Proanthocyanidin Content

The proanthocyanins content of the carob extracts was determined by the procedure described by Quiroz-Reyes et al. [34]. In 1.5 mL of solution A (n-BuOH/HCl, 95:5, *v*/*v*), 250 μL of carob extract (1 mg/mL) and 50 μL of solution B (2% NH_4_Fe(SO_4_)_2_·12H_2_O in 2M HCl) were added and thoroughly mixed. The mixture was then heated for 1 h at 95 °C in a water bath and cooled immediately in ice. The absorbance was measured at 550 nm using a Shimadzu UV-1700 spectrometer (Tokyo, Japan). The blank value was prepared in the same way as the samples and subtracted from all of them. The quantification was performed using a calibration curve of cyanidin chloride (0.001–0.25 mg/mL) as a standard. The results were expressed as cyanidin chloride equivalents per g extract dw (CyE/g dw).

### 2.6. Determination of the Radical Scavenging Activity on DPPH

The effect of the extracts on DPPH radical was estimated, according to Sarikurkcu et al., with some modifications [35]. The radical scavenging ability of the extracts was measured from the bleaching of the purple colored methanol solution of DPPH. A total of 1 mL of various concentrations (0.001–1 mg/mL) of the carob extracts was added to 1 mL of freshly made DPPH radical solution, 0.2 mM in methanol and shaken vigorously. A control sample was prepared containing 1 mL of H_2_O instead of the extract solution. The mixture was allowed to stand for 30 min in a dark at room temperature and the absorbance was measured at 517 nm using a Shimadzu spectrophotometer UV-1700 (Tokyo, Japan). The inhibition of free radical DPPH in percent (I%) was calculated in following way:

$$I\%=100\times \frac{\left(A_{\mathrm{control}}-{\;A}_{\mathrm{sample}}\right)}{A_{\mathrm{control}}}$$

where A_control_ is the absorbance of the control reaction using the control sample, and A_sample_ is the absorbance of the extract.

The antioxidant activity was measured by calculation of the EC_50_ value using OriginPro 2021 software (OriginLab Corporation, Northampton, MA, USA) [36]. The EC_50_ value represents the quantity of the antioxidant required to produce half of the response (DPPH radical scavenging). Antiradical curves were plotted referring the logarithm of extracts concentration on the x axis and their inhibition percentage calculated on the y axis, forming a sigmoidal dose–response graph with a standard four-parameter formula expressed as:

$$y=\;A1+\frac{A2-A1}{1+10^{\left({\mathrm{logx}}_{0}-x\right)p}}$$

where A1 is the bottom asymptote (baseline) and A2 the top asymptote, which represents the maximum response; p is the hill slope of the curve and x_0_ is the concentration at the center of the curve (inflection point), also recognized as the EC_50_ value. The antioxidant activity was also expressed as the Trolox equivalent antioxidant capacity (TEAC), by calculating the EC_50_ value of the Trolox standard (expressed in μg/mL) and dividing it with the EC_50_ value of each sample (expressed in μg/mL) [37].

### 2.7. High Performance Liquid Chromatography (HPLC) Analysis

The HPLC analysis of polyphenolic extracts was carried out using a Thermo Scientific—Dionex Ultimate 3000 UHPLC system equipped with a diode array detector (Thermo Fisher Scientific, Walthman, MA, USA), based on the method reported previously by Bittova et al. [38]. The separation was performed on an Acclaim 120 C18 column (4.6 × 150 mm, 5 μm particle size) (Thermo Fisher Scientific, Walthman, MA, USA) and a C18 guard column (4.6 × 10 mm, 5 μm particle size) (Thermo Fisher Scientific, Walthman, MA, USA) thermostated at 25 °C. The mobile phases were eluent A, acetic acid/water (1/99, *v*/*v*), eluent B, methanol and eluent C, acetonitrile. The flow rate was 0.7 mL/min and the gradient was as follows: 0–10 min, 70% A, 25% B, 5% C; 10–15 min, 65% A, 25% B, 10% C; 15–50 min, 45% A, 40% B, 15% C and then the initial solvents were maintained for another 5 min for column re-equilibration. The Ultraviolet-Visible (UV-VIS) absorbance spectra were monitored between 190 and 400 nm. The sample injection volume was 10 μL. Prior to the injection of the standard solutions and the samples to the HPLC system, they were filtered through MCE syringe filters (0.20 μm pore size, JG Fenneran, Vineland, NJ, USA). The individual phenolic compounds were identified by comparing the retention times and UV-VIS absorbance spectra with their corresponding analyzed standards. The quantification of gallic acid and cinnamic acid, identified as the two most abundant phenolic compounds in our samples, was performed at 280 nm using the external standard calibration method. Instrument control, data acquisition and evaluation were performed with the Chromeleon 7 Chromatography Data System software (Thermo Fisher Scientific, Walthman, MA, USA). Additional information on the HPLC method (Table S1) and chromatograms (Figure S1) are available as Supplementary Materials S1.

### 2.8. ATR-FTIR Spectroscopy

The ATR-FTIR spectra were obtained with a Vertex 70 FTIR spectrometer (Bruker Optics, Ettlingen, Germany), equipped with a single-reflection ZnSe ATR accessory (Pike Technologies, Madison WI, USA) and a DTGS detector (Bruker Optics, Ettlingen, Germany). Approximately 10 μL of 10 mg/mL extract solution was deposited on the crystal and allowed to dry for ~50 min until a film layer was formed prior to the measurement. A background spectrum against air was recorded before each sample spectrum. The background and sample spectra were acquired at 4 cm^−1^ resolution with 40 scans from 4000 to 400 cm^−1^. Each spectrum is the average of spectra from at least three different samples. The ATR-FTIR spectra were normalized employing vector normalization. The Opus 7.0 software (Bruker Optics, Ettlingen, Germany) was used for spectra collection and analysis.

### 2.9. Statistical Analysis

The results are presented as the mean ± standard deviation of quadruplicates. The error bars in all figures correspond to the standard deviations. Statistical tests were performed using the OriginPro 2021 software (OriginLab Corporation, Northampton, MA, USA). The experimental data were tested for the normal distribution and homogeneity of variances (Levene’s test) and then subjected to the analysis of variance (ANOVA). One-way ANOVA was applied to the experimental data to determine the significance of the effects (roasting temperature and time) on TPC, proanthocyanidin content, gallic acid content, cinnamic acid content and antioxidant activity (EC_50_) of carob extracts. The significant differences among the means were estimated through Tukey’s honestly significant difference (HSD) test. Pearson correlations were performed to assess the relationships among the different parameters studied. For all statistical analysis, *p* < 0.05 was considered as statistical significance.

## 3. Results and Discussion

### 3.1. Polyphenolic Content and Antioxidant Activity

The two-step solid–liquid extraction procedure followed in this work, enabled the production of polyphenol-rich carob powder extracts with total polyphenolic content (TPC) of 85 mg GAE/g extract dw, which corresponded to 0.504 g GAE/100 g of raw (unroasted) carob powder. This TPC value is within the range of those previously reported [11,12,15,17]. It is noted that all photospectrometric assays, chromatographic analyses and FTIR characterization were performed on the extracts without further processing, and all results were quoted per gram of extract dw.

**Figure 1:**
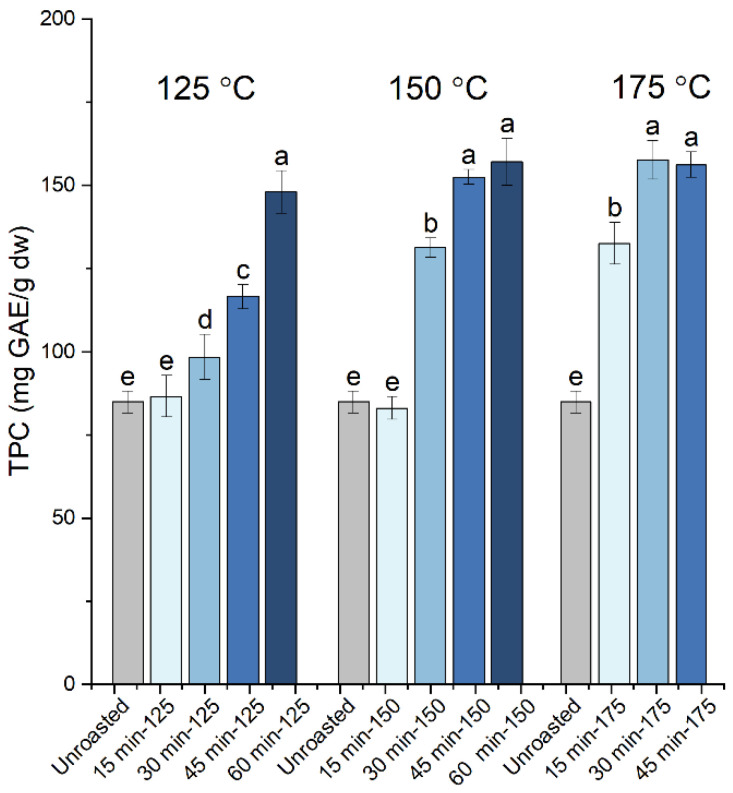
The total polyphenolic content (TPC) of the carob powder extracts obtained prior to (unroasted) and after the roasting of kibbles at 125 °C (for 15, 30, 45 and 60 min), 150 °C (for 15, 30, 45, 60 min) and 175 °C (for 15, 30, 45 min). The different letters show the statistically significant differences according to Tukey’s honestly significant difference (HSD) test at *p* < 0.05.

The changes in the TPC of the carob powder extracts upon the roasting of the kibbles at 125 °C (for 15, 30, 45 and 60 min), 150 °C (for 15, 30, 45, 60 min) and 175 °C (for 15, 30, 45 min) are shown in Figure 1 and summarized in Table 1. The lowest value for the TPC was recorded for the extract from raw carob powder and from the samples roasted at 125 °C and 150 °C for the shorter roasting time of 15 min. All other roasted samples displayed an increase in the TPC. For the roasting temperature of 125 °C, the TPC increased gradually with the increasing roasting time to reach a maximum value of 148 mg GAE/g dw at 60 min. For the roasting temperatures of 150 °C and 175 °C, a similar maximum plateau value exceeding 150 mg GAE/g dw was reached at 45 min and 30 min, respectively, and remained largely the same for the longer roasting times. The maximum TPC value in roasted samples reached a 1.9-fold increase relative to the unroasted sample. The increase in the TPC upon roasting can partially be attributed to the improved solubility of polyphenols, due to the breakdown of cellular structures and their release from decomposing polymeric structures, processes that are promoted during roasting. However, since the Folin–Ciocalteu reagent is reactive towards other reducing compounds besides phenols, MRPs also contribute to the observed increase in TPC [11,12]. Previous studies have also reported an increase in the TPC of roasted carob powder versus the raw samples, although there are deviations in the effects of time and roasting temperature. Sahin et al. [11] reported that the heat-induced changes in carob powder, including the increase in TPC, particularly accelerate between 20 and 60 min of roasting at all tested temperatures (135 °C, 150 °C and 165 °C), while Cepo et al. [12] stated that the majority of processes occurred during the first 15 min of roasting at similar tested temperatures (130 °C, 150 °C and 165 °C). Our data demonstrated that both the roasting temperature and time affected the TPC, leading to a similar maximum plateau value at the temperatures examined in this work (125 °C, 150 °C and 175 °C), albeit at different roasting times. The rates of the processes leading to the increase in TPC varied depending on the roasting temperature.

**Figure 2:**
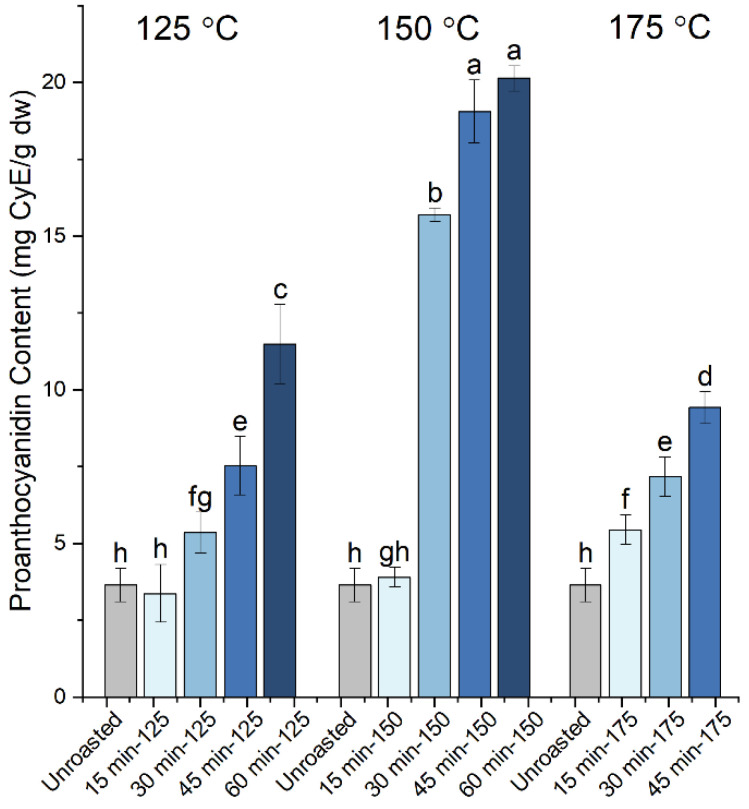
Proanthocyanidin content of the carob powder extracts obtained prior to (unroasted) and after the roasting of kibbles at 125 °C (for 15, 30, 45 and 60 min), 150 °C (for 15, 30, 45 and 60 min) and 175 °C (for 15, 30 and 45 min). The different letters show the statistically significant differences according to Tukey’s HSD test at *p* < 0.05.

To investigate the effect of roasting on different polyphenolic compounds, we also photometrically determined the proanthocyanidin content of the extracts from raw and roasted carob kibbles. Figure 2 shows the changes in the proanthocyanidin content of the extracts from carob powder upon roasting at the indicated times and temperatures. Similar to the TPC, the lowest content in proanthocyanidins was recorded for the extract from the raw carob powder at the value of 3.6 mg CyE/g dw. The proanthocyanidin content increased with the roasting time, reaching a maximum at 60 min and, specifically, a value of 11.5 mg CyE/g dw at 125 °C (3.2-fold increase), 20.1 mg CyE/g dw at 150 °C (5.5-fold increase) and 9.4 mg CyE/g dw at 175 °C (2.6-fold increase). Therefore, the data revealed that varying maximum proanthocyanidin content can be obtained at different roasting temperatures, with 150 °C being the optimum temperature for enhancing the content of extracts. Overall, we suggest that roasting enhances the solubility and extractability of proanthocyanidins, while the competing thermal degradation of proanthocyanidins can be responsible for the lower values observed at 175 °C in comparison to 150 °C.

**Figure 3:**
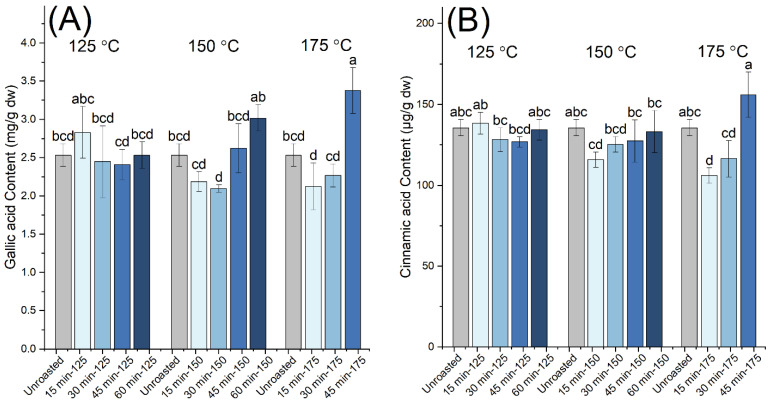
(**A**) Gallic acid and (**B**) cinnamic acid contents of the carob powder extracts obtained prior to (unroasted) and after the roasting of kibbles at 125 °C (for 15, 30, 45 and 60 min), 150 °C (for 15, 30, 45 and 60 min) and 175 °C (for 15, 30 and 45 min). The different letters show the statistically significant differences according to Tukey’s HSD test at *p* < 0.05.

Moreover, we monitored the gallic and cinnamic acid contents of the extracts from raw and roasted carob kibbles (Figure 3 and Table 1), since they were the most abundant free monomeric phenolic compounds identified by the employed HPLC method in our samples. Gallic acid was the dominant compound, in agreement with previous studies [14,17,20]. Unlike the TPC and proanthocyanidin content, moderate variations were observed for the gallic acid content between the raw and roasted samples, and the variations in cinnamic acid content were even subtler. A motif of decrease and subsequent increase in the content of the monomeric phenolic compounds with the increasing time of roasting was identified at the intermediate and highest roasting temperatures used in this work (150 °C and 175 °C). This motif was attributed to the competing processes of thermal degradation, leading to decreasing content, versus the release of the monomers from their bound and conjugated forms, leading to increasing content. For gallic acid, the highest contents were observed in the samples roasted at 150 °C for 60 min and 175 °C for 45 min, suggesting that, under these roasting conditions, the rate of release of gallic acid from its bound and conjugated forms becomes dominant over the thermally-induced degradation reactions.

**Figure 4:**
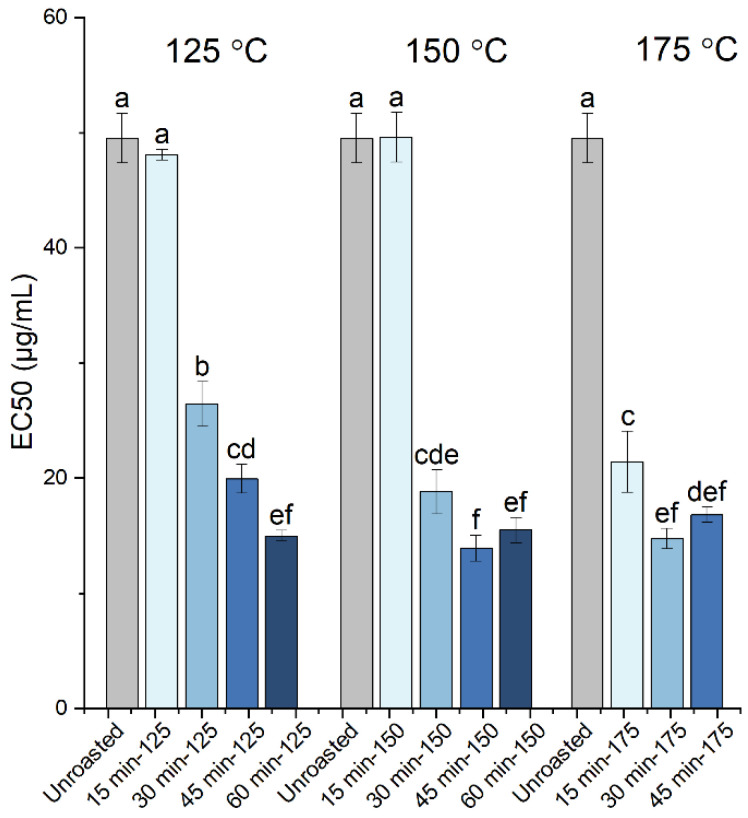
DPPH scavenging activity (expressed as EC_50_) of the carob powder extracts obtained prior to (unroasted) and after roasting of kibbles at 125 °C (for 15, 30, 45 and 60 min), 150 °C (for 15, 30, 45 and 60 min) and 175 °C (for 15, 30 and 45 min). The different letters show the statistically significant differences according to Tukey’s HSD test at *p* < 0.05.

The radical scavenging activity of the carob powder extracts was evaluated by the DPPH assay with the determination of the EC_50_ values (half maximal effective concentration), representing the amount of extract required to produce half of the response (scavenging of the DPPH radical). The EC_50_ values determined for the extracts from raw and roasted carob kibbles, are presented in Figure 4 and Table 1. The highest EC_50_ value of 49.5 μg/mL was obtained for the extract from raw carob powder, while the lowest EC_50_ values and, thus, the highest antioxidant activity, were observed for the extracts from the samples roasted for 60 min at 125 °C, for 45 and 60 min at 150 °C and for 30 and 45 at 175 °C (up to a 3.3-fold increase in antioxidant activity). Overall, the EC_50_ values determined for the extracts in this work were significantly lower compared to those reported previously for the extracts from raw carob kibbles (33.26 g/L) and roasted carob products (7.04–9.96 g/L) [17], suggesting that the extracts obtained, herein, were of a higher antioxidant capacity. Moreover, the extracts from both the raw and roasted carob samples all had EC_50_ values below 50 μg/mL, demonstrating an overall strong antioxidant capacity, as also confirmed by the calculation of the TEAC values (included in Table 1). The Pearson correlation matrix among the antioxidant activity (EC_50_), TPC, proanthocyanidin, gallic and cinnamic acid contents (Table 2) revealed that the antioxidant activity was strongly associated with the TPC, while the correlation with the proanthocyanidin content was lower and no correlation existed with the phenolic monomers content. Since polyphenols and MRPs contribute to the TPC, we suggest that both the release of bound phenolics and formation of MRPs are important contributors to the enhancement of the antioxidant activity upon roasting. A better understanding of the link between antioxidant activity and the chemical/structural changes induced upon roasting requires a technique that is sensitive to the molecular structure and, thus, we also employed FTIR spectroscopy in this work.

### 3.2. ATR-FTIR Characterization

**Figure 5:**
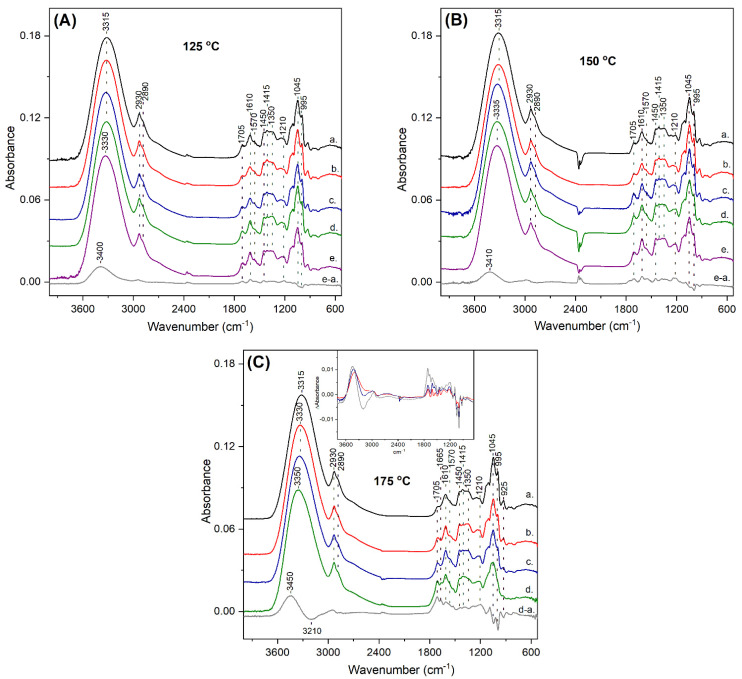
ATR-FTIR spectra of carob powder extracts obtained prior to (trace a) and after the roasting of kibbles for 15 min (trace b), 30 min (trace c), 45 min (trace d) and 60 min (trace e) at the indicated temperatures in each panel: (**A**) 125 °C, (**B**) 150 °C and (**C**) 175 °C. The spectra in all panels have been translated along the vertical axis for clarity. The difference FTIR spectrum of the extract from the longer roasting time minus the unroasted sample (trace e−a in panels (**A**,**B**) and trace d−a in panel (**C**)) is included in all panels. The inset in panel (**C**) shows the FTIR difference spectra: b−a (red), c−a (blue) and d−a (grey) in an overlay plot.

FTIR spectroscopy is a valuable tool in the identification of the molecular structure of compounds as well as the chemical changes taking place upon the roasting of carob kibbles, since FTIR spectra comprise of bands characteristic of chemical bonds [39]. In this work, the ATR-FTIR approach was employed as a versatile sampling method requiring minimal sample preparation prior to the spectral measurements. Figure 5 shows the ATR-FTIR spectra of the polyphenolic extracts from raw (trace a in all panels) and roasted carob kibbles (traces b–e in all panels). The ATR-FTIR spectra of the extracts obtained from unroasted carob kibbles (trace a in all panels) displayed a major broad band at 3315 cm^−1^, attributed primarily to the O–H stretching vibration of hydroxyl groups from phenolic compounds and carbohydrates, with some possible contribution from the N–H stretch [39,40,41]. The frequency and bandwidth of the 3315 cm^−1^ band indicated intermolecular hydrogen bonding interactions for the hydroxyl groups [39]. The bands at 2930 cm^−1^ and 2890 cm^−1^ originated from the C–H stretching vibrations of sp^3^ C–H bonds. The band at 1705 cm^−1^ was assigned to the CO stretching mode demonstrating the presence of carbonyl groups. The vibration at 1610 cm^−1^ with the shoulder at 1570 cm^−1^ resulted from overlapping bands, including those arising from C=C–C aromatic ring bonds, C=N and C=C bonds, while contributions from amide or carboxylate groups are also possible in this spectral region [32,39,40,41]. In the 1450–1300 cm^−1^ region, multiple bands were observed, which were mainly due to CH and OH bending vibrations. The intensity at 1210 cm^−1^ was partially attributed to the C–O (phenol) stretch and the prominent bands at the ~1150–990 cm^−1^ region were assigned to C–C and to C–O stretches from alkyl ester, ether, methoxy and alcohol groups [32,39,40,41].

The analysis of the ATR-FTIR spectra of carob kibbles extracts revealed their complex chemical nature, since the vibrations of various classes of compounds were detected, with the phenolic compounds being indisputably identified. The antioxidant activity of polyphenolic extracts is primarily exerted by the phenolic hydroxyl groups, which displayed a strong contribution in the ~3300 cm^−1^ region in our spectra. In particular, the antioxidant activity can be related to the number and position of phenolic hydroxyls, while the methoxy and alkyl ester groups are among the functional groups that frequently appear in a wide range of phenolic compounds and affect the antioxidant activity [42]. The contributions of such groups were identified in the ATR-FTIR spectra of the carob kibbles extracts, in this study.

Figure 5 includes the ATR-FTIR spectra of the extracts, after the roasting of carob kibbles at 125 °C (A), 150 °C (B) and 175 °C (C) for 15 min (trace b), 30 min (trace c), 45 min (trace d) and 60 min (trace e). The thermal processing of the carob kibbles resulted in frequency shifts and changes in the relative intensity of various bands in the ATR-FTIR spectra of the extracts, which depended on the roasting temperature and duration. As shown in Figure 5A, the changes in the ATR-FTIR spectra of the roasted samples were subtle and mainly observed at the spectrum corresponding to 60 min roasting time (trace e). The most readily observed change involved the upshift of the broad hydroxyl band from 3315 cm^−1^ to 3330 cm^−1^. The upshift of the O–H bond demonstrated weaker hydrogen bonding interactions of the OH groups in the extracts from roasted samples relative to that from unroasted kibbles. It is well established that the intermolecular hydrogen bonding interactions of the hydroxyl groups weaken the O–H bond and lead to the downshift of the O–H stretch [39]. To identify even more subtle changes in the ATR-FTIR spectra upon roasting, we utilized the difference spectroscopy approach since, in the difference spectra, a decrease in the intensity of a vibration will appear as a negative peak and an increase as a positive one. Therefore, the difference ATR-FTIR spectrum of 60 min roasting minus the unroasted sample (trace e–a) was calculated and is shown in Figure 5A. This difference spectrum demonstrated a broad positive band at 3400 cm^−1^ attributed to the presence of an increased number of phenolic hydroxyl groups with weak intermolecular hydrogen interactions in the roasted samples. This observation was also consistent with the enhanced release of polyphenolic compounds from the complex fruit matrix upon roasting, and the observed increased TPC and proanthocyanidin content in the extracts from roasted samples relative to that from unroasted kibbles. Moreover, other positive bands appeared in the difference spectrum in the C=O region (1750–1600 cm^−1^), while negative bands were observed in the C–O region (1100–990 cm^−1^). It is interesting to note that the decrease in the intensity of C–O single bonds versus the increase in C=O double bonds has been recently reported as a characteristic feature in the FTIR spectra observed upon the formation of melanoidins in the Maillard reaction of different carbohydrates [41]. Common features were also identified in the comparison with the ATR-FTIR spectra of high molecular weight cocoa melanoidins that were recently reported [32]. We suggest that the formation of melanoidins was the source for the observations in the fingerprint region in our spectra. The melanoidins produced in different food matrices have been reported to positively correlate to antioxidant activity and to have the potential to scavenge reactive α-dicarbonyls [28,29,34,43].

The ATR-FTIR spectra of the extracts from the carob kibbles roasted at 150 °C are shown in Figure 5B. A slight upshift of the 3315 cm^−1^ hydroxyl band was already observed at 30 min roasting time (trace c) and reached 3335 cm^−1^ at 45 min and 60 min, as depicted in traces d and e, respectively. The difference spectrum of 60 min roasting minus the unroasted sample (trace e–a) revealed the presence of a broad positive band at 3410 cm^−1^, comparable to that observed for 125 °C with a slightly higher frequency. The positive peaks in the C=O region (1750–1600 cm^−1^) and the negative bands in the C–O region (1100–990 cm^−1^) were observed, similar to the corresponding difference spectrum of the 125 °C sample, with some intensity differences.

Figure 5C includes the ATR-FTIR spectra obtained for the extracts from the carob kibbles roasted at 175 °C. At this temperature, the changes in the ATR-FTIR spectra appeared at earlier roasting times as it was evident in the 15 min spectrum (trace b), in which the hydroxyl band shifted at 3330 cm^−1^, while it shifted further to 3340 cm^−1^ and 3350 cm^−1^ in the 30 min (trace c) and 45 min (trace d) spectra, respectively. The spectrum corresponding to the 45 min roasting time (trace d) demonstrated the most extensive changes, including significant intensity changes and the formation of new peaks. The difference spectrum of 45 min roasting minus the unroasted sample (trace d–a), indeed, revealed diverse features relative to the difference spectra of 125 °C and 150 °C. In the 3600–3000 cm^−1^ region, a peak to through pattern was observed at 3450/3210 cm^−1^. Moreover, multiple intense positive peaks were detected in the carbonyl region with a prominent newly formed vibration at 1665 cm^−1^. Because the changes in the ATR-FTIR spectra were readily observed, even at short roasting times at 175 °C, we have included the difference spectra for all the roasting times at this temperature as an inset, in Figure 5C. The difference spectra corresponding to the samples roasted for 15 min (red trace) and 30 min (blue trace) at 175 °C, resembled those obtained for 60 min roasting time at 125 °C and 150 °C. The formation of additional carbonyl groups that was observed when roasting was performed at 175 °C for 45 min was not associated with further changes in the antioxidant activity. Therefore, the products formed in this sample can include contaminant compounds that are more likely to accumulate at higher temperatures, as shown in previous studies [14,15].

Overall, the upshift of the O–H stretch in the ATR-FTIR spectra of the roasted samples, partially attributed to the phenolic hydroxyl, indicated a significant change in the intermolecular hydrogen bonding interactions of the phenolic hydroxyl groups at all roasting temperatures, thereby providing a possible contribution for the increased antioxidant activity upon roasting. Hydrogen bonding interactions are considered as important factors of antioxidant performance. Intermolecular hydrogen bonding contributes to diminished antioxidant activity, while intramolecular hydrogen bonding lowers the O–H bond dissociation enthalpy of polyphenols and, consequently, enhances antioxidant activity [42]. Our ATR-FTIR data indicated the presence of an increasing number of hydroxyl groups with weakened intermolecular hydrogen interactions in the roasted samples. Consequently, both the increased quantity of polyphenols due to the improved extractability, as well as the weakening of the intermolecular hydrogen bonding of the phenolic hydroxyls, are suggested to be crucial for the enhancement of antioxidant activity upon roasting. Except for the changes involving the polyphenolic compounds in the roasted extracts, the Maillard reaction is also a contributor in the enhancement of antioxidant activity. Our ATR-FTIR data indicated the formation of MRPs, and the spectral features of these products were consistent with the characteristic IR signatures of melanoidins.

## 4. Conclusions

In summary, for the first time, the present study utilized ATR-FTIR spectroscopy to explore the structure–antioxidant activity relations in roasted carob kibbles. The structural changes involving the hydrogen bonding interactions of the phenolic hydroxyl groups were detected and considered as contributors to the increased antioxidant activity of carob powder extracts upon roasting, in addition to the enhanced extractability of phenolic compounds from the fruit matrix. The competitive effects arising from the thermal degradation of polyphenolic compounds at the highest roasting temperature of 175 °C used in this study was evidenced, particularly for proanthocyanidins. The FTIR detection of melanoidins signatures in the roasted samples, which displayed the highest antioxidant activities, laid the basis for the further investigation of these bioactive compounds in carob. Experiments for the separation of the low and high molecular weight fractions of roasted carob kibbles are underway in our laboratory, to allow for a comprehensive characterization of the structure and antioxidant activity of the low molecular weight compounds and of the polymeric melanoidins.

## Figures and Tables

**Table 1:** Total polyphenolic content (TPC), proanthocyanidin content, gallic acid content, cinnamic acid content, EC_50_ and Trolox equivalent antioxidant capacity (TEAC) values of the extracts from unroasted and roasted carob powder samples at the indicated roasting conditions.

Roasting Conditions		TPC (mg GAE/g dw)	Proanthocyanidins (mg CyE/g dw)	Gallic Acid (mg/g dw)	Cinnamic Acid (μg/g dw)	EC_50_ (μg/mL)	TEAC
Unroasted		84.9 ± 3.3 ^e^	3.64 ± 0.55 ^h^	2.53 ± 0.15 ^bcd^	135.5 ± 5.0 ^abc^	49.5 ± 2.2 ^a^	0.23 ± 0.01
125 °C	15 min	86.6 ± 6.1 ^e^	3.37 ± 0.93 ^h^	2.83 ± 0.34 ^abc^	138.3 ± 6.8 ^ab^	48.1 ± 0.5 ^a^	0.24 ± 0.00
	30 min	98.4 ± 6.8 ^d^	5.36 ± 0.67 ^fg^	2.45 ± 0.47 ^bcd^	128.2 ± 7.3 ^bc^	26.5 ± 1.9 ^b^	0.43 ± 0.03
	45 min	116.5 ± 3.6 ^c^	7.53 ± 0.95 ^e^	2.41 ± 0.20 ^cd^	126.8 ± 3.1 ^bcd^	20.0 ± 1.2 ^cd^	0.56 ± 0.03
	60 min	148.0 ± 6.5 ^a^	11.49 ± 1.29 ^c^	2.53 ± 0.17 ^bcd^	134.3 ± 6.4 ^abc^	15.0 ± 0.5 ^ef^	0.77 ± 0.03
150 °C	15 min	83.1 ± 3.5 ^e^	3.90 ± 0.32 ^gh^	2.19 ± 0.13 ^cd^	115.8 ± 4.7 ^cd^	49.6 ± 2.2 ^a^	0.23 ± 0.01
	30 min	131.3 ± 2.9 ^b^	15.69 ± 0.21 ^b^	2.10 ± 0.05 ^d^	125.4 ± 4.8 ^bcd^	18.8 ± 1.9 ^cde^	0.55 ± 0.03
	45 min	152.5 ± 2.3 ^a^	19.05 ± 1.02 ^a^	2.62 ± 0.32 ^bcd^	127.3 ± 13.1 ^bc^	13.9 ± 1.1 ^f^	0.79 ± 0.03
	60 min	157.1 ± 7.2 ^a^	20.12 ± 0.42 ^a^	3.02 ± 0.18 ^ab^	133.1 ± 13.0 ^bc^	15.5 ± 1.1 ^ef^	0.72 ± 0.03
175 °C	15 min	132.5 ± 6.2 ^b^	5.44 ± 0.49 ^f^	2.13 ± 0.31 ^d^	106.0 ± 4.8 ^d^	21.4 ± 2.7 ^c^	0.47 ± 0.04
	30 min	157.7 ± 5.9 ^a^	7.17 ± 0.65 ^e^	2.27 ± 0.15 ^cd^	116.4 ± 11.3 ^cd^	14.7 ± 0.9 ^ef^	0.75 ± 0.03
	45 min	156.2 ± 3.9 ^a^	9.42 ± 0.51 ^d^	3.38 ± 0.30 ^a^	155.9 ± 14.0 ^a^	16.8 ± 0.7 ^def^	0.66 ± 0.02

Data are presented as the mean ± standard deviation of quadruplicates. The different letters show the statistically significant differences between the roasting conditions according to Tukey’s HSD test at *p* < 0.05.

**Table 2:** Pearson correlation matrix among the total polyphenolic content (TPC), proanthocyanidin content (PC), gallic acid content (GA), cinnamic acid content (CIA) and antioxidant activity expressed as EC_50_ values.

	TPC	PC	GA	CIA	EC_50_
**TPC**	**1**	**0.71042**	0.31998	0.09808	**−0.91444**
**PC**		**1**	0.21168	0.1464	**−0.67768**
**GA**			**1**	**0.84864**	−0.12447
**CIA**				**1**	0.00249
**EC_50_**					**1**

Correlations in bold are significant at *p* < 0.05.

## Data Availability

Data is contained within the article and Supplementary Material.

## Supplementary Materials

The following are available online at https://www.mdpi.com/article/10.3390/antiox10122025/s1, Figure S1: (A) chromatogram of the standard phenolic compounds recorded at 280 nm: gallic acid (1), pyrogallol (2), catechin (3), epicatechin (4), caffeic acid (5), p-coumaric acid (6), ferulic acid (7), myricetin (8), cinnamic acid (9), quercetin (10) and kaempferol (11). (B) Chromatogram of carob powder extract recorded at 280 nm, Table S1: retention times, regression equations, coefficients of determination (R^2^), LODs, LOQs and % relative standard deviation (%RSD) of the peak areas.
