# Supplementary material for: Linking the Dynamic Changes in the In Vitro Antioxidant Activity of Carob Kibbles upon Roasting to the Chemical and Structural Changes Revealed by FTIR Spectroscopy
Source: Antioxidants (Basel). 2021 Dec 20;10(12):2025. doi: 10.3390/antiox10122025 (PMC8698282; doi:10.3390/antiox10122025)
Supplement: Supplementary file 1 [file antioxidants-10-02025-s001.zip › antioxidants-1482263-supplementary.pdf]

Supplementary Materials

Linking the Dynamic Changes in the In Vitro Antioxidant Activity of Carob Kibbles Upon Roasting to the Chemical and Structural Changes Revealed by FTIR Spectroscopy

Anna Marina Grigoriou, Eftychia Pinakoulaki *

Department of Chemistry, University of Cyprus, Nicosia 1678, Cyprus

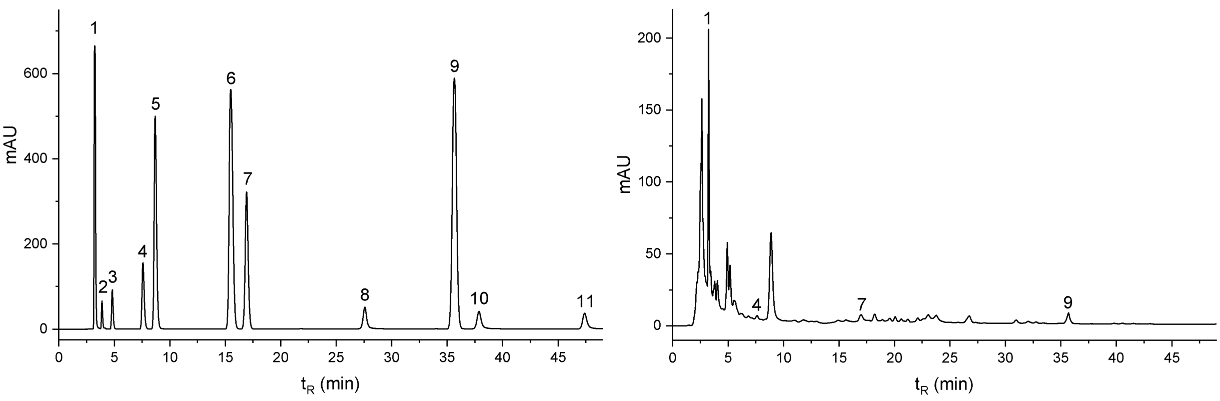

(A) **(B)**

Figure S1: (A) Chromatogram of the standard phenolic compounds recorded at 280 nm: gallic acid (1), pyrogallol (2), catechin (3), epicatechin (4), caffeic acid (5), p-coumaric acid (6), ferulic acid (7), myricetin (8), cinnamic acid (9), quercetin (10) and kaempferol (11). **(B)** Chromatogram of carob powder extract recorded at 280 nm.

It is noted that phenolic compounds in the carob extracts were identified by comparison of retention times and UV-Vis absorbance spectra with their corresponding analyzed standards. We reported the contents of gallic acid and cinnamic acid in the carob powder extracts, the two most abundant monomeric phenolic compounds identified by the employed HPLC method, whose concentrations were well above LOQs in all analyzed samples, with gallic acid evidently being the dominant one.

Table S1: Retention times, regression equations, coefficients of determination (R^2), LODs, LOQs and % relative standard deviation (%RSD) of the peak areas.

Compound	Regression Equation for Calibration Curve*	t_R (min)	R^2	LOD (μg/mL)	LOQ (μg/mL)	Intra-day precision RSD_{area} (%)**	Inter-day precision RSD_{area} (%)***
Gallic Acid	y = 0.5978x - 0.0259	3.2	0.999	0.15	0.45	0.96	1.05
Pyrogallol	y = 0.03x - 0.0018	3.9	1	0.58	1.74	1.10	1.74
Catechin	y = 0.1617x - 0.0397	4.8	0.999	0.80	2.42	1.28	3.18
Epicatechin	y = 0.1881x - 0.0261	7.6	0.999	0.33	1.01	1.17	1.02
Caffeic Acid	y = 0.772x - 0.1419	8.7	1	0.63	1.91	1.21	1.17
p-Coumaric Acid	y = 1.2919x - 0.303	15.5	1	0.13	0.41	1.10	0.95
Ferulic Acid	y = 0.6709x + 0.0076	17.0	1	0.22	0.66	1.14	0.95
Myricetin	y = 0.6824x - 0.3334	27.6	0.999	1.08	3.28	1.28	2.56
Cinnamic Acid	y = 1.8332x + 0.0876	35.7	1	0.18	0.53	1.06	1.06
Quercetin	y = 0.7581x - 0.1999	37.9	0.999	0.25	0.76	1.61	1.03
Kaempferol	y = 0.8648x - 0.1415	47.4	0.999	0.22	0.66	1.17	0.99

* For all compounds calibration curves were constructed from peak areas at 280 nm except for myricetin, quercetin and kaempferol at 370 nm

** Five replicates within the same day

*** Three replicates per day for four different days
